# The way a nose could affect pregnancy: severe and recurrent epistaxis

**DOI:** 10.11604/pamj.2019.34.49.19558

**Published:** 2019-09-24

**Authors:** Laura Giambanco, Vito Iannone, Maddalena Borriello, Giuseppe Scibilia, Paolo Scollo

**Affiliations:** 1 Ob&Gyn Department, S. Antonio Abate Hospital, Trapani, Italy; 2 Ob&Gyn Department Cannizzaro Hospital, Catania, Italy

**Keywords:** Epistaxis, pregnancy, anaemia

## Abstract

Massive and severe epistaxis is an uncommon event in pregnancy. It could be life threatening and could affect the normal pregnancy course. The best management is still on debate; it could be medical, conservative or surgical. Pregnancy termination often is problem solving. Hormonal changes during pregnancy affects nasal physiology. Vaginal delivery, labour induction or cesarean section are all suitable, after hemodynamic stabilization of pregnant woman. We report a case and review the available literature.

## Introduction

El Goulli *et al.* in 1979 [1] reported on a case of severe epistaxis in pregnancy with maternal collapse and fetal death. From then on few case reports have been published) [1-9] (Table 1) with different management of epistaxis in third trimester of pregnancy. Furthermore, the mode of delivery, vaginally or by cesarean section, varies in this short report, probably due to qualitative and quantitative levels of blood loss. Epistaxis is a common problem in pregnancy, secondary to an increased nasal mucosa vascularity. A large volume epistaxis is uncommon in women without risk factors (such as anticoagulants therapy or blood clotting disorders) [9]. When severe blood loss through the nose occurs in third trimester of pregnancy there are different therapeutical approaches: nose packing, hemostatic foam/sponge, cautery or diathermy, ligation of sphenopalatine artery. The acute blood loss could be life threatening both for mother and fetus. Hospitalization is mandatory, blood transfusion is often required, tranexamic acid intravenous therapy, ENT specialist (ear, nose, throat) intervention to manage epistaxis. Then, obstetricians have to decide if it's necessary to induce labour and if it's possible vaginal delivery. Theoretically, vaginal delivery could be contraindicated because of the efforts required, the pushes during the late second stage, Valsalva maneuvers may induce epistaxis again. In literature it's reported only one case of vaginal delivery, by Hardy *et al.* [6], in the other cases an emergent or elective cesarean section was performed. Fetal anemia could be secondary to acute massive maternal epistaxis refractory to conservative management. In few cases, pregnancy termination represents the best solution, because maternal changes in volemia and nasal mucosae stops at the same time.

**Table 1:** Care reports published

Year, 1st Author	N° pts	Management epistaxis	Management pregnancy
1974, Green	1	Local pressure, nasal packing	Emergency CS
1979, El Goulli	1	Nasal packing	VD
1985, Howard	1	Nasal packing, bipolar diathermy, external carotid artery ligation, nasal balloon	Emergency CS
1995,Brathwaite	1	Nasal packing and balloon	Emergency CS
2002, Cooley	1	Nasal packing and balloon	Emergency CS
2008, Hardy	1	Nasal packing, bipolar diathermy, artery ligation	Vaginal delivery
2013, Cornthwaite	1	Nasal packing, bipolar diathermy	Elective CS
2014, Crunkhorn	1	Nasal packing, SPA ligation, bipolar cautery and diathermy	Elective CS
2019, Piccioni	1	Nasal packing bipolar cautery, iv acid tranexamic	Emergency CS

CS: cesarean section; VD: vaginal delivery; IV: intravenous; SPA: sphenopalatine artery

## Patient and observation

A 26 years old nulliparous woman presented to our observation for severe right-sided epistaxis, non self-limiting. She had few episodes in the previous two weeks of epistaxis, even 3-4 per day, usually stopping spontaneously. She reported a negative ENT consultation except for mild hyperemia of nasal mucosa. The pregnancy was unremarkable, considered at low risk, at 39.6 weeks of gestation. Her familiar and personal history was negative for blood coagulopathies and hypertension. We tried to stop severe epistaxis with nasal packing and intravenous tranexamic acid. On the basis of the failure of our procedures an otolaryngologist performed an endoscopy identifying bleeding enlarged vessels. Nasal packing with hemostatic sponge was successful, the pregnant woman was admitted to ob&gyn department. Her hemoglobin levels dropped down to 6 mg/dl and she needed 4 red cell packs. The day after her right nostril despite the tampons started bleeding again. She was referred to otolaryngologist who packed again the nose, bilaterally and used hemostatic glue. After 2 more days she started bleeding again, her nose was again packed with glue and tampons and further 2 red cell packs were given. The obstetric scan revealed biometry at 40^th^ centile, the Bishop score was 5, so we decided to induce labour after obtaining her informed written consent. The labour induction was performed with intravaginal prostaglandins (10 mg dinoprostone). After 18 hours the labour seemed to proceed well with cervical dilatation of 6 cm, level of head 0, but the cardiotocography (CTG) trace revealed anomalies that after 1 hour induced the shift versus cesarean section. No epistaxis happened during labour nor during cesarean section. Two days after nasal tampons were removed, the patient was discharged the day after. One month later the woman reported general good health and no epistaxis episodes, nor mild nor massive.

## Discussion

Epistaxis in pregnancy is not so uncommon and self-limiting, is more than three-times than in non-pregnant women [10]. However, when it occurs in the third trimester it could become life-threatening and a severe deal for obstetricians. Operative ENT (ear, nose, throat) management is generally requested, even if there is limited evidence regarding the correct approach of severe epistaxis in pregnancy, complicated by the evidence that certain products are contraindicated (bismuth iodophorm paraffine gauzes, soaked ribbon gauzes) (Table 2) [8]. When termination of epistaxis is achieved, the further management depends on gestational age. When maternal and fetal conditions are good enough to wait, conservative management is to be preferred, in order to avoid preterm birth risks, but if the gestational age is near to term, it's evaluable delivery induction or elective cesarean section. Delivery induction is possible when cervix is favourable, using vaginal prostaglandins or oxytocin infusion, if Bishop score is 0-3 a planned cesarean section is to be preferred.

**Table 2:** Acute epistaxis in pregnancy: management options (8, modified)

Tranexamic acid iv
Cautery/diathermy
Anterior nasal packing
Paraffin gauzes
Posterior nasal packing
Anti-hemostatic matrix
Sphenopalatine artery ligation (general anesthesia)
Anterior ethmoidal artery ligation (general anesthesia)
Posterior ethmoidal artery ligation (general anesthesia)
Radiological embolization (unquantified risks)

## Conclusion

Hormonal changes during pregnancy affects nasal physiology; oestrogens cause vascular congestion, mucosal oedema and recurrent rhinitis (rhinitis of pregnancy) [11-13] in 20% of pregnant women. Estrogens could also have indirect effects on vascular wall by regulating NO signaling pathway (i.e. VEGF, VEGFR-2) [8]. Meanwhile progesterone provokes an increase of blood volume, which could cause an augmentation of vascular changes with severe epistaxis, in the absence of coagulative disorders or organic nasal abnormalities (polyps). Even the placenta contributes to the risk of epistaxis, releasing placental growth hormone that determines systemic effects such as vasodilatation. Last but not least immunological changes could lead to nasal hypersensitivity [11]. When severe epistaxis occurs in pregnancy a multidisciplinary approach is recommended, by obstetricians, ENT specialists [8]. The management could be conservative or surgical. The goal is to obtain control of blood loss in order to going on safely the pregnancy or to plan an elective cesarean section or labour induction.

## Competing interests

The authors declare no competing interests.
